# Digital Health Education for the Future: The SaNuRN (Santé Numérique Rouen-Nice) Consortium’s Journey

**DOI:** 10.2196/53997

**Published:** 2024-04-30

**Authors:** Julien Grosjean, Frank Dufour, Arriel Benis, Jean-Marie Januel, Pascal Staccini, Stéfan Jacques Darmoni

**Affiliations:** 1 Department of Biomedical Informatics, Rouen University Hospital, Rouen, France; 2 Laboratory of Medical Informatics and Knowledge Engineering in e-Health (LIMICS), INSERM U1142, Sorbonne Université, Paris, France; 3 URE Risk Epidemiology Territory INformatics Education and Health (RETINES), Université Côte d’Azur, Nice, France; 4 Department of Digital Medical Technologies, Holon Institute of Technology, Holon, Israel

**Keywords:** digital health, medical informatics, education, health education, curriculum, students, teaching materials, hybrid learning, program development, capacity building, access to information, e-learning, open access, open data, skills framework, competency-based learning, telemedicine training, medical simulation, objective structured clinical examination, OSCE, script concordance test, SCT, virtual patient

## Abstract

*Santé Numérique Rouen-Nice* (SaNuRN; “Digital Health Rouen-Nice” in English) is a 5-year project by the University of Rouen Normandy (URN) and Côte d’Azur University (CAU) consortium to optimize digital health education for medical and paramedical students, professionals, and administrators. The project includes a skills framework, training modules, and teaching resources. In 2027, SaNuRN is expected to train a significant portion of the 400,000 health and paramedical students at the French national level. Our purpose is to give a synopsis of the SaNuRN initiative, emphasizing its novel educational methods and how they will enhance the delivery of digital health education. Our goals include showcasing SaNuRN as a comprehensive program consisting of a proficiency framework, instructional modules, and educational materials and explaining how SaNuRN is implemented in the participating academic institutions. SaNuRN is aimed at educating and training health and paramedical students in digital health. The project is a cooperative effort between URN and CAU, covering 4 French departments. It is based on the *French National Referential on Digital Health* (*FNRDH*), which defines the skills and competencies to be acquired and validated by every student in the health, paramedical, and social professions curricula. The SaNuRN team is currently adapting the existing URN and CAU syllabi to *FNRDH* and developing short-duration video capsules of 20-30 minutes to teach all the relevant material. The project aims to ensure that the largest student population earns the necessary skills, and it has developed a 2-tier system involving facilitators who will enable the efficient expansion of the project’s educational outreach and support the students in learning the needed material efficiently. With a focus on real-world scenarios and innovative teaching activities integrating telemedicine devices and virtual professionals, SaNuRN is committed to enabling continuous learning for health care professionals in clinical practice. The SaNuRN team introduced new ways of evaluating health care professionals by shifting from a knowledge-based to a competencies-based evaluation, aligning with the Miller teaching pyramid and using the Objective Structured Clinical Examination and Script Concordance Test in digital health education. Drawing on the expertise of URN, CAU, and their public health and digital research laboratories and partners, SaNuRN represents a platform for continuous innovation, including telemedicine training and living labs with virtual and interactive professional activities. SaNuRN provides a comprehensive, personalized, 30-hour training package for health and paramedical students, addressing all 70 *FNRDH* competencies. The project is enhanced using artificial intelligence and natural language processing to create virtual patients and professionals for digital health care simulation. SaNuRN teaching materials are open access. It collaborates with academic institutions worldwide to develop educational material on digital health in English and multilingual formats. SaNuRN offers a practical and persuasive training approach to meet the current digital health education requirements.

## Introduction and Background

Digital health and health informatics are at the crossroads of medicine and health sciences, computer science and engineering, information and communication sciences, mathematics, statistics, technology, and innovation management [1]. Digital health has been a component of regular training in medical schools for 40 years [2-4] under different labels, such as medical informatics [5], medical computing (in the United States) [6], and e-health [7], with high heterogeneity in content at the national level. In France, digital health is a subdomain of public health, which is also part of the training in medical and paramedical schools [8].

In 2022, the French Ministry of Health, and in particular its Delegation of Digital Health, published an open call for project proposals to support innovative approaches to develop initial academic and continuing professional education in digital health to health-related students, professionals and administrators; law specialists; computer scientists; and data protection officers. “Health-related students and professionals” was mainly referring to students enrolled in health-related programs, including medicine; odontology; pharmacy; midwifery; and paramedical fields such as nursing, physiotherapy, speech therapy, and hearing-aid technician, as well as training programs for social workers [9]. A budget of €71 million (US $75.73 million) has been secured to achieve this specific call to deal with the expected need to train over 400,000 health and paramedical professions students in 2027 at the national level.

The University of Rouen Normandy (URN) and the Côte d’Azur University (CAU), as a consortium, have successfully answered this call by getting a 5-year grant for their joint project, *Santé Numérique Rouen-Nice* (SaNuRN; “Digital Health Rouen-Nice” in English) [10]. SaNuRN began on September 1, 2022, with a cost estimate of €6,891,923 (US $7,351,441) and a grant contribution of €3,951,200 (US $4,214,646), with the goal of training around 30,000 students by 2027.

Before the initiation of this national project in France, there was a notable deficiency in digital health training for health students and practically none in paramedical schools. The primary focus was on health students pursuing master’s degrees, such as medicine, pharmacy, dentistry, and midwifery. For instance, a national master’s program in medical informatics has been established at Sorbonne University for the past 25 years. However, up until 2020, there was no existing digital health training curriculum for health students at the bachelor’s degree level. Consequently, a comprehensive curriculum in digital health had to be developed from scratch for both health and paramedical students at the bachelor’s degree level.

Before the SaNuRN project, a 10-hour module was introduced for all first-year medical students in CAU in 2020, and in URN, a 20-hour module was implemented for some first-year medical students in 2021. The open call from the Delegation of Digital Health at the French Ministry of Health emphasized allocating 80% of the training effort to the bachelor’s degree level. One of the challenges of the SaNuRN project was assembling a team of digital health specialists to train all health-related students. The initial 2 years of the SaNuRN project (2022-2024) were dedicated to implementing a digital health teaching module for all health-related students, including both health and paramedical programs, at the bachelor’s degree level.

This paper aims to provide an overview of the SaNuRN project, highlighting its pedagogical innovations and how its implementation will optimize digital health education. Our objectives are to present SaNuRN as a whole, comprising a skills framework, training modules, and teaching resources, and to describe how SaNuRN is and will be deployed in the consortium institutions. Below, we describe the SaNuRN project and its objectives. Next, we detail the skills framework and training modules, explaining the teaching resources and how they are deployed. Finally, we discuss the pedagogical innovations and expected impact of the SaNuRN project on digital health education and the quality of care and patient outcomes.

## Building a Digital Health Education Lifelong Platform (SaNuRN) as a Cooperation Achievement

### Overview

The SaNuRN project emerged in the context of a long-lasting cooperation between URN and CAU in *digital health* (SJD and PS), *medical simulation* (Professors Louis Sibert and Jean-Paul Fournier), and *general practice* (Professors Matthieu Schuers and David Darmon), as a primary use case for teaching digital health during postgraduate studies and residency. From our perspective, this extensive cooperation was a decisive factor in the grant application’s success. URN is located in the northwest of France and CAU is located in the southeast. The distance between them is around 1000 km, and this points out the challenges related to the SaNuRN consortium, which is well managed by using as much dematerialized infrastructure as possible to deliver digital health teaching and learning content. Thus, the first-stage objective of SaNuRN is to educate and train all students in health-related fields in digital health at 4 French departments (Seine-Maritime and Eure in Normandy for URN, and Alpes-Maritimes and Var in Provence-Alpes-Côte d’Azur for CAU), to cover a population of 4 million inhabitants; the target of SaNuRN is to train about 2800 health-related students each year.

### Targeted Skills and Competencies

To support this effort, the SaNuRN team activities are based on the *French National Referential on Digital Health* (*FNRDH*) [11]. Created and published in 2021, this referential gives a framework and defines the skills and competencies to be acquired and validated by every student in the health, paramedical, and social professions curricula [8]. The exhaustive list of skills and competencies of the *FNRDH* is detailed in Multimedia Appendix 1. The skills defined in the *FNRDH* are organized into five competency categories: (1) security, (2) health data, (3) communication in health, (4) digital tools in health, and (5) telehealth and teleactivities. *FNRDH* is built around a three-level hierarchy: (1) the 5 competencies as introduced above, (2) a total of 25 subcompetencies (eg, to identify an end user or a health professional and to characterize and manage nominative data, applying the European rules such as the General Data Protection Regulation [GPDR]), and (3) a total of 70 different abilities (eg, to understand the life cycle of the digital health data and to take actions against virus and malware). In June 2023, *FNRDH* was integrated [12] into the HeTOP terminology server [13] to create a Catalog and Index of Health Digital Teaching Resources (CIDHR) [14-16] to be usable by all French health and paramedical students.

### Adapting Existing Resources to the FNRDH Framework

Since the beginning of the project in October 2022, the primary need has consisted of adapting the existing URN and CAU syllabi to *FNRDH*. The SaNuRN project builds in a matrix format to adapt *FNRDH* for each degree (bachelor’, master’s, doctorate, or residency) and each field of study (eg, medicine, nursing, or physiotherapy). Furthermore, the course focuses on digital health in each field of study and, at each degree level, is limited to 30 hours of lectures and practices to address the 70 competencies of the *FNRDH* skills framework. To manage this challenge, the SaNuRN team is developing short-duration video capsules of 20-30 minutes to teach all the relevant material adapted to the degree levels and fields of study by taking into account the expectations within each degree, discipline, and potential learning sites (URN, CAU, and their partners).

It is essential to notice that the pedagogical components of the SaNuRN project are derived from existing teaching resources previously developed by the 2 Departments of Digital Health at URN and CAU. For example, West Normandy has 7 nursing schools (partners of URN), and the SaNuRN project has adapted its training to each of them specifically.

**Figure 1.**
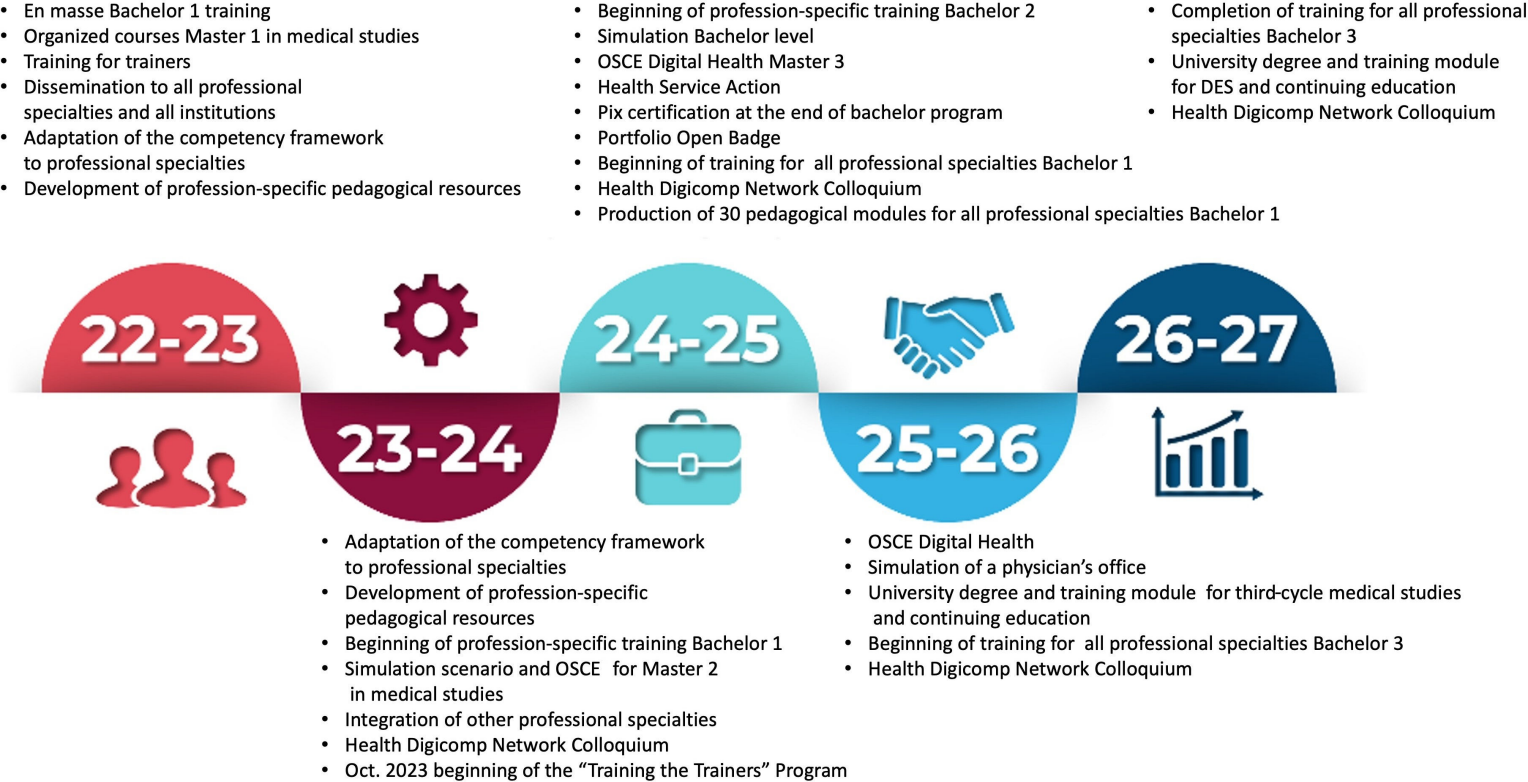
Overall schema of the SaNuRN project timeline. DES: Diplôme d'Etudes Spécialisées (Residency Program); OSCE: Objective Structured Clinical Examination; SaNuRN: Santé Numérique Rouen-Nice (Digital Health Rouen-Nice).

Since the inception of the SaNuRN project, additional teaching modules have been introduced to address the list of *FNRDH* skills and competencies. Among these modules, one is dedicated to cybersecurity and another to health data. This supplementary course emphasizes practical applications, featuring instructional videos on the use of specific tools aligned with the skills and competencies of the *FNRDH*. These tools include (1) a secure email tool, (2) guidance on accessing Mon Espace Santé—a digital platform designed for citizens and patients to manage their digital documents actively, (3) Health French National Identification, and (4) ethics in health (refer to Figure 1). The SaNuRN consortium used existing videos from the French National Digital Health Agency to develop these new teaching resources.

As of January 2024, the personalized 30-hour module is accessible in 2 modes: as freely available teaching resources for any health-related student through an open data website [17] and as specific video capsules within the URN and CAU private teaching environments. A total of 24 hours of preexisting resources, predating the SaNuRN project, were adapted to cater to various audiences, focusing on nurses and pharmacists. Overall, 80% (56/70) of the *FNRDH* competencies are covered by at least 1 SaNuRN teaching resource [17].

In line with the strategic decision made by the SaNuRN consortium in response to the French Delegation of Digital Health, all teaching materials generated during the project will be openly accessible on a website (“teaching open data”) [17]. Furthermore, from these teaching materials (eg, cybersecurity), several short-duration capsules were developed to suit the specific needs of students in various specialties, including medicine, pharmacy, and nursing.

A significant advantage of SaNuRN is that all these resources are freely accessible to everyone, aiming to benefit health and paramedical students and professionals.

### Facilitating Digital Health Education Adoption and Improvement

To ensure that the largest student population earns the necessary skills, the SaNuRN project has developed a 2-tier system. The first tier involves selecting teaching staff in each professional specialty field involved in the project that will act as facilitators. They will enable the efficient expansion of the project’s educational outreach and facilitate periodic updating of the skills framework within each professional specialty. Additionally, these facilitators will support the students in learning the needed material efficiently. The training of these facilitators began in May 2023 for a year.

Based on the outcomes of the first tier, the second tier will consist of adjusting the educational resources that were initially only based on the *FNRDH*. This will facilitate the deployment, student and teaching staff engagement, and adoption of the digital health teaching modules in all health and paramedical specialties.

### The SaNuRN Approach to Digital Health Education Innovation

#### Overview

As a part of the requirements of the SaNuRN’s grant, 80% of the funding is dedicated to first-degree students (bachelor’s), corresponding to most of the health and paramedical students that are enrolled. The SaNuRN consortium has already largely fulfilled this objective by massively educating and training in traditional classroom settings or through self-training.

In the next 3 and half years (see Figure 1), the SaNuRN consortium will focus on pedagogical digital health innovations for the second and third degrees of all health-related fields of study. For example, the consortium has already planned pluriprofessional training sessions for the first semester of 2024 (eg, medicine residents with nursing students, both involved in specific teleconsultations). The first scheduled training session is about clinical data warehouses from various health and paramedical perspectives.

New paradigms are already present in the SaNuRN digital health syllabus and have been introduced to public health residents, particularly the paradigm of “One Digital Health” [18], defined as the intersection of *one health* and *digital health*. The SaNuRN team has developed two other innovations: (1) modification of the evaluation process with a shift from a knowledge-based to a competencies-based evaluation, as proposed by the Miller teaching pyramid [19], such as the Objective Structured Clinical Examination, and (2) Script Concordance Test in digital health, using a Health Professional Connected Office (see Figure 1).

Furthermore, several aspects will be mainly at the heart of the innovation of the SaNuRN project, as presented below.

#### Interactive and Innovative Components of Courses

The characteristics of the SaNuRN project are primarily the combination of knowledge and expertise from URN, CAU, and their public health and digital research laboratories and partners.

#### Integrated Telemedicine Devices

Two medical simulation centers, at URN [20] and at CAU [21], have already established living labs. These labs include simulated professional offices and patient apartments, providing a platform to test various software in different health situations, especially in general practice. Soon, 2 integrated telemedicine units will be available for health and paramedical students to test different health situations using scenarios of simulated patients.

By 2027, the SaNuRN project aims to implement several teaching modules that will be financially self-sustainable (ie, that will run in the future without the financial support available for the grant period). These modules will offer digital health training for continuous learning in general practice and private companies, including big pharma and health technology (or “medtech”) companies. For instance, a full-day teaching module has been developed to help private companies handle clinical data warehouses. The medical simulation centers in Rouen (URN) and Nice (CAU) will be used to conduct most of these training sessions.

#### Living Labs With Virtual and Interactive Professional Activities

The SaNuRN program includes a conversational virtual clinical simulator using artificial intelligence techniques combined with natural language processing, with the modeling of clinical situations defined for the training of all health care professionals (eg, physicians, pharmacists, nurses, and physiotherapists).

Analogous to expert systems, this tool, on the one hand, will be able to play the role of a professional (ie, backward chaining), asking questions to a patient while adjusting to the patient’s responses (similarly to a computer-assisted diagnostic aid). On the other hand, the system will play the role of the patient (ie, forward chaining), answering a professional’s questions (simulated clinical examination of the virtual patient).

## A Comprehensive Overview of SaNuRN’s First Achievements

### Digital Health Education Before SaNuRN (September 2022)

Before SaNuRN (September 2022), digital health was already taught in URN and CAU. Indeed, for example, first-year students at the health schools at URN and CAU received an initial and primary education on digital health. Specifically, since 2021, a total of 15% (150/1000) of the students at URN took a 20-hour course as a part of a minor in health digital science, and since 2020, a total of 100% (1000/1000) at CAU received a 10-hour mandatory course. These digital health courses are performed in a traditional large classroom setting at URN and CAU.

### Adaptations in Nursing Schools

In West Normandy, a teaching self-learning module was provided to the 7 nursing schools, representing 600 nursing students. In the first semester of 2022, the teaching module was directly derived from the one provided for health students, with an identical duration of 20 hours. Very quickly, in response to the feedback from nursing students, the SaNuRN consortium analyzed the teaching discrepancies between the real needs of these nursing students and the content of the digital health teaching module. Therefore, a 6-hour training was reorganized for the second semester. Furthermore, for each teaching module, all the examples provided were modified and adapted to the nursing student’s needs (eg, to demonstrate the need for health smart cards in their specific practice).

### Implementation and Expansion During SaNuRN’s First Year

During the first academic year of the SaNuRN project (2022-2023), around 2000 students specializing in health care and paramedical specialties were trained. According to our knowledge and the various national agencies involved in the program, this number is significantly higher than those at other French universities. The West Normandy nursing schools provided only 20 hours of digital health training. These schools have requested an additional 10-hour course in the third year of the curriculum to fulfill the 30-hour teaching requirement.

### Expansion and Hybrid Learning

In the ongoing academic year (2023-2024), the SaNuRN consortium has engaged several new student cohorts. At URN, 200 second-year medical students (ie, students who passed the first highly selective year at the school of health and chose to study medicine) have participated in hybrid training, including 4 hours of face-to-face organized courses and 25 hours of self-training (see Figure 1). Additionally, around 100 second-year pharmacy students (ie, similar to students who chose to study medicine, except they chose to study pharmacy instead) may opt for this digital health teaching module. A total of 100 physiotherapy and ergotherapy students are also involved in the project, dedicating 24 hours to self-training. For all these new URN students, the SaNuRN team proposes the following module: a 2-hour introduction teaching (see Figure 1).

At CAU, 400 nursing students from 4 nursing schools will participate in the SaNuRN project in 2024. Lastly, 20 public health residents from URN and CAU will have access to advanced teaching resources. Thus, 100% of medical and paramedical students will be trained in digital health at both universities in 2025.

The Delegation of Digital Health of the French Ministry of Health aims to educate and train 400,000 students in digital health by 2027 using a 30-hour module based on the *FNRDH* guideline. The SaNuRN project plans to teach 13,200 students over 5 years.

The overall SaNuRN project during the 5 years is summarized in Figure 1; most of the effort is made for students in the first degree of their studies to attain the 100% rate of trained students in digital health. The SaNuRN consortium will fit this goal in June 2025. Then, specific contents are available for second and third degrees to improve the knowledge and competencies in specific situations and medical and paramedical disciplines (eg, videos and live demonstrations of teleconsulting with nurses, physicians, or physiotherapists).

### Training Trainers

#### Goals and Framework

Since May 2023, specific training sessions have been performed for digital health trainers. The “Training the Trainers” component of the SaNuRN project is instrumental to attaining the project’s primary goal, that is, providing education on digital health for undergraduate students of all medical, paramedical, and social disciplines in the academic year 2024-2025. The goals of this component are to obtain from the trainees—who all are faculty members actively teaching in the various academic programs and institutions responsible for undergraduate education in medicine, paramedicine, and social work—the most accurate information about their students such as their profiles, schedules and course works, and preparation for the discipline of digital health.

#### Program Structure and Implementation

This information is further used to:

- Design the most appropriate pedagogical resources in terms of format, depth of knowledge, types of learning activities, and modes of assessment.
- Evaluate and train the faculty members in the discipline of digital health.
- Train them in the design of e-learning curricula and the use of digital pedagogical resources.
- Prepare them for the integration of the 30 hours of education to digital health in their respective programs.

#### Core Activities and Learning Objectives

The “Training the Trainers” program has been organized as a yearlong, ongoing, asynchronous, and remote training activity primarily to respond to the significant disparity regarding the trainees’ availability, who, for the most part, could not commit to a fixed time slot, and to allow for an extensive immersion within the discipline itself and consistent exposure to digital technologies. All the collaborative and remote tools used in the development and course of this program were unknown to the trainees, and it took time and practice for all of them to attain a good level of proficiency and confidence.

This model allowed the program to admit new trainees at different stages and moments of its development.

#### Development of Pedagogical Resources

The program started in May 2023 with 15 faculty members enrolled. It is scheduled to last until the end of May 2024, with, as of today, 34 members representing all the disciplines concerned with the integration of new courses in digital health.

The core activity of the program consists of the complete understanding of the reference framework (*FNRDH*; Multimedia Appendix 1) and the planning and design of its integration within existing courses and academic programs. Through a collective explication of all capacities included in the *FNRDH*, the group of trainees has identified 5 learning topics forming the core common foundation shared by all health-related disciplines: “cybersecurity,” the “digital health system,” “digital communication,” “digital professional communication,” and “further developments of digital health.” In order to accommodate the various specificities of the existing academic programs, each of these topics has been divided into teaching modules of roughly 20 minutes, allowing easy customization and integration into existing curricula.

All program trainees contribute to designing and producing these 30 modules, representing the first 10 hours of education in digital health for first-year undergraduate students in health-related disciplines. These modules are designed to be delivered as autonomous self-teaching, asynchronous modules, thus allowing all faculty members to monitor students’ activity and progress with their own methods and tools.

Together with these modules, the trainees are producing web-based interactive resources with the help of a partner of the SaNuRN program, IKIGAI, a nonprofit game design company. Two types of such interactive resources are currently being produced: a gamified quiz and a set of flashcards for practice and memorization.

#### Innovative Pedagogical Tools: Introduction of Learning and Assessment Scenario

The “Training the Trainers” program provides trainees with a fully immersive experience in digital communication and education and an in-depth analysis of the *FNRDH*, allowing them to clearly envision the multiple ramifications of the new discipline of e-health.

With the “Training the Trainers” program (see Figure 1), the SaNuRN project has introduced, at the undergraduate level, one major innovative pedagogical tool, the Learning and Assessment Scenario. This tool consists of a detailed outline of a complex professional situation involving digital tools and technologies and the collaboration of professionals from other disciplines. The students presented with this situation must engage in collaborative activities to assess the situation’s multiple dimensions and propose a coordinated plan of action to solve the issue. This teaching tool prefigures tools used at the graduate and postgraduate levels, such as the Objective Structured Clinical Examination and Script Concordance Test. The Learning and Assessment Scenario also serves as an efficient tool to teach the much-needed interprofessional collaboration skills that are brought to higher levels of complexity and depth by digital technologies. With this progressive strategy, the educational program created by SaNuRN, covering the 3 cycles of medical, paramedical, and social work studies, creates a consistent continuum of educational engagement for faculty members and students in meaningful interactions with digital technologies.

### Evaluation Plans

Currently, no formal (qualitative or quantitative) evaluation has been performed in the SaNuRN project; 2 qualitative evaluations have already been planned: in URN and CAU, 1 for medical students and 1 for nurse students. One indirect positive measure is the presence of health-related students in URN and CAU during the first year’s training sessions. However, the presence was not mandatory, and over 90% of health-related students were present in the 20-hour training module in URN and 10-hour training module in CAU.

## Discussion

### Overview

During the first academic year of the SaNuRN project (2022-2023), around 2000 students specializing in health care and paramedical specialties received training, a significantly higher number than other French universities. In the ongoing academic year (2023-2024), various new student cohorts are participating in the project, including medical, pharmacy, physiotherapy, and ergotherapy students and public health residents. The SaNuRN project aims to educate 13,200 students over 5 years, contributing to the Delegation of Digital Health’s goal of training 400,000 students by 2027.

The project primarily focuses on first-degree students in the initial years (bachelor’s), with specific content for second- and third-degree students (master’s and PhD or residency) to enhance knowledge and competencies in various medical and paramedical disciplines.

The SaNuRN consortium plans to introduce innovative teaching methods, including interprofessional training sessions, competency-based evaluations, and the use of telemedicine devices. Interactive and innovative course components, combined with living labs and virtual clinical simulators, form the core of the project’s innovations.

The key strengths and limitations of the SaNuRN project rely on (1) the fulfillment of the French Ministry of Health’s aim to make digital health learning mandatory and (2) compliance with professional international recommendations, even when the specificities for the French higher education system make it challenging.

### Strengths

#### Fulfilling National Commitments With the SaNuRN Project

By September 2024, learning digital health will be mandatory for all health and paramedical students in France. At that time, the SaNuRN project will be able to fulfill the national commitment to teaching digital health by addressing the 70 *FNRDH* competencies in a 30-hour training package.

#### Fitting Global Trends in Digital Health Education

In addition to France, several countries are proposing digital health training at the national level. However, only a handful of countries have established such competencies for clinical practice in their core medical school curriculum [22]. In England, the National Health Service has launched the Digital Readiness Education program [23]. It aims to improve digital skills, understanding, knowledge, and awareness across the multidisciplinary health and care workforce to support new working methods. This program focuses on continuous training. A qualitative study evaluated digital competencies in Singapore for its national medical school curriculum (which included 4 medical schools) [22]. One of the main conclusions was the need to enhance the sharing of educational resources and expertise. This point is also crucial in the French program, so the SaNuRN project has decided to create “open access” and “open data” teaching resources that are shareable with all French health and paramedical schools. An experiment was also conducted in Italy, using Petri-Nets to improve digital health literacy [24].

#### Looking at Internationalizing the SaNuRN’s Concept

An essential advantage of SaNuRN is that all teaching materials created during the project are freely available to anyone, even explicitly targeting all health and paramedical students and professionals [17]. Most of the teaching material is created in French. However, thanks to international collaborations with institutions such as the Holon Institute of Technology (HIT) in Israel, a large part of the SaNuRN material is coproduced and available in English. This approach allows these partners, URN and CAU, to use the relevant SaNuRN resources in their curricula. For example, some SaNuRN resources (lessons) have been cocreated or coenhanced with lecturers in charge of health data science courses of the Department of Digital Medical Technologies at the HIT in Israel [25].

#### Complying With the International Medical Informatics Association Recommendations

The International Medical Informatics Association has published 2 versions of its international recommendations in biomedical and health informatics education, initially in 2000 and revised in 2010 and 2023 [26]. The International Medical Informatics Association recommendations are a framework for national initiatives in biomedical and health informatics education and for constituting international programs and exchange of students and teachers in this digital health field. Zainal et al [27] have proposed a scoping review on clinical informatics training in medical school education curricula; these authors proposed 4 main recommendations that are very similar to those used in the SaNuRN project: situating digital health curriculum within specific contexts, developing evidence-based guidelines for robust digital health education, developing validated assessment techniques to evaluate curriculum effectiveness, and equipping educators with relevant digital health training.

### Limitations

#### Needing to Align With Other International Standards

The teaching model may not be entirely compatible with other international approaches; drawing inspiration from experiences in other countries and attempting to fit within a shared framework would be advisable.

#### Temporarily Focusing on Undergraduate Students

Because the project was principally focusing its efforts on undergraduate degrees for its first 2 years, no international collaboration was initiated, apart from a cooperation with the HIT in Israel, as such collaboration usually targets graduate and postgraduate levels. At the national level, for the master’s and PhD degrees, the SaNuRN consortium is planning to cooperate in 2024 with several French universities (Sorbonne Université, Paris Cité, Besançon Université, and Rennes Université), as well as European universities, in particular University for Health Sciences, Medical Informatics and Technology in Austria and continuing its cooperation with the HIT in Israel.

## Conclusion

The SaNuRN project addresses France’s national commitment to teaching digital health. SaNuRN addresses all 70 *FNRDH* skills and competencies (Multimedia Appendix 1) with a comprehensive, personalized, 30-hour training package for each health or paramedical student according to their degree level, field of study, and university curriculum. This innovative approach is enhanced by using artificial intelligence and natural language processing to create virtual patients and professionals for digital health care simulation, allowing each student to replay and practice various clinical situations. SaNuRN teaching materials are openly accessible. Moreover, SaNuRN, aiming to answer new needs of the French health schools and paramedical professions, is collaborating with academic institutions worldwide to develop educational material in digital health in English and multilingual formats. SaNuRN offers an enhanced training approach that is both effective and persuasive, making it a challenging solution to the current digital health education requirements in France and potentially Europe and worldwide.

## Supplementary material

10.2196/53997 Multimedia Appendix 1 List of skills and competencies of the *French National Referential on Digital Health* (*FNRDH*).

## Abbreviations

CAU: Côte d’Azur University
CIDHR: Catalog and Index of Health Digital Teaching Resources
FNRDH: French National Referential on Digital Health
GPDR: General Data Protection Regulation
HIT: Holon Institute of Technology
SaNuRN: Santé Numérique Rouen-Nice (Digital Health Rouen-Nice)
URN: University of Rouen Normandy
