# Supplementary material for: Digital Health Education for the Future: The SaNuRN (Santé Numérique Rouen-Nice) Consortium’s Journey
Source: JMIR Med Educ. 2024 Apr 30;10:e53997. doi: 10.2196/53997 (PMC11082434; doi:10.2196/53997)
Supplement: Multimedia Appendix 1 [file mededu-v10-e53997-s001.pdf]

Multimedia Appendix 1: List of skills and competencies of the French National Referential on Digital Health (FNRDH)

1. Health Data

1.1 Identifying a user or a healthcare professional

1.1.1 Understanding the issues and criteria related to identity vigilance towards a user [National Health Identifier (INS), national reference frameworks for the identity of individuals]

1.1.2 Understanding the issues and criteria related to the identification of a professional or an establishment [Shared Directory of Healthcare Professionals (RPPS) for individual professionals, The National Directory of Health and Social Establishments (FINESS) for establishments and legal entities]

1.2 Characterizing and processing personal health data in compliance with regulations

1.2.1 Understanding Law No. 78-17 on information technology, files, and freedoms of January 6, 1978 (LIL) and EU Regulation 2016/679 on general data protection of April 27, 2016 (GDPR)

1.2.2 Defining health data in the context of GDPR and knowing how to distinguish personal data, anonymous data, and pseudonymous data

1.2.3 Understanding the life cycle of digital health data

1.2.4 Knowing the regime for the processing of personal health data

1.2.5 Distinguishing processing methods applied to health data from processing methods applied to personal data and the sanctions incurred

1.2.6 Knowing how to remain compliant with GDPR

1.3 Accessing health data in compliance with professional and legal requirements

1.3.1 Identifying criteria for accessing user data: understanding the regulatory framework and the concept of a care team

1.3.2 Knowing the specifics of health data storage [National Health Data System (SNDS) and health data warehouses]

1.3.3 Knowing what a cloud, a host, and the Health Data Hub (HDH) are

1.3.4 Knowing the criminal and civil liabilities incurred

1.3.5 Respecting professional secrecy applied to medical and healthcare professions, the protection of professionals and users, and shared medical confidentiality

1.3.6 Respecting the rights of users and obligations related to patient information: knowing how to behave and knowing how to do

1.3.7 Using tools to access user data [National Health Identifier (INS), Digital Health Professional Card (CPS), e-CPS identifier, Professional Training Card (CPF), Pro Health Connect]

1.3.8 Exhibiting ethics in the use of health data

1.4 Leverage health data for evaluation, research, and innovation

1.4.1 Knowing the major issues related to artificial intelligence, algorithms, biases, and related ethical principles

1.4.2 Knowing major institutes and research programs

1.4.3 Knowing the specific policy for processing data for research purposes (Research Involving Human Subjects, Jardé Law, GDPR, LIL)
1.4.4 Searching for published scientific data and knowing how to interpret them, producing relevant bibliography
1.4.5 Sharing data and participating in a scientific research or innovation program

2. Cybersecurity

2.1 Designing and maintaining a secure digital work environment

2.1.1 Knowing cybersecurity reference frameworks [especially the General Policy for the Security of Information Systems (PGSSI) and the ANSSI Computer Hygiene Guide]
2.1.2 Securing physical access points (session locking)
2.1.3 Configuring workstations and mobile phones (managing antivirus and updates, data encryption and backup, using software compliant with security and confidentiality rules)
2.1.4 Managing removable devices and nomadic use of equipment
2.1.5 Knowing different authentication principles, the importance of strong and two-factor authentication, and managing strong passwords
2.1.6 Securing email and following best practices for sending and receiving emails
2.1.7 Understanding the challenges of electronic identification applied to the health sector
2.1.8 Implementing best practices to secure digital environment

2.2 Protecting against and responding to incidents

2.2.1 Knowing the different types of malicious actions
2.2.2 Securing internet browsing, knowing how to identify trustworthy sites
2.2.3 Knowing how to protect against viruses and malicious actions
2.2.4 Identifying a personal data breach within the GDPR framewok
2.2.5 Responding to health cybersecurity incidents

3. Communication in Health practice

3.1 Using adequate tools to interact with users for effective information sharing

3.1.1 Knowing the regulatory framework governing good communication practices with all stakeholders [National Health Identity (INS), medical confidentiality, codes of ethics...]
3.1.2 Mastering the concept of professional responsibility
3.1.3 Identifying digital communication methods (MSSanté, MSS-C)

3.2 Interacting adequately among professionals, with the user, with caregivers and companions, and with institutions and administrations

3.2.1 Knowing the governance and actors of digital health in France (DNS, ANS, GRADeS, CNIL, CERT santé, ...)
3.2.2 Adopting professional behavior
3.2.3 Adopting ethical principles
3.2.4 Identifying and addressing a situation of digital precariousness or a digital-related disorder
3.2.5 Informing, sensitizing, and supporting students and interns
3.2.6 Providing information to the user in their procedures
3.2.7 Adopting good practices and tools to interact and share health data
3.2.8 Using digital services of institutions and administrations

3.3 Interacting on the internet while managing one's digital identity

3.3.1 Knowing the issues related to e-reputation (referencing, defamation, ...)
3.3.2 Knowing best practices while connecting to platforms
3.3.3 Interacting as a healthcare professional
3.3.4 Managing and deleting a digital identity

4. Digital Tools in Health Practice

4.1 Mastering professional software and digital services

4.1.1 Being able to define e-health
4.1.2 Identifying professional tools (software and platforms) constitutive of the professional ecosystem (Bundle of Services for Health Professionals [BSP])
4.1.3 Being aware of the e-health market and major digital French transformation programs digital (e.g. digital Segur, Hop'en, digital ESMS, ...)
4.1.4 Knowing the responsibility associated with medical decisions
4.1.5 Navigating through legal notices, property rights, and user rights
4.1.6 Identifying functionalities and tools necessary for practice (interoperability, security, health data exchange, backup, storage, signature, SaaS or On-Premise)

4.2 Using a connected device or a mobile application and analyzing their reliability

4.2.1 Knowing the different categories of connected objects and health applications [Higher Health Authority (HAS) frameworks, categorization, ...]
4.2.2 Identifying digital tools tailored to the expectations and healthcare needs of users, as well as the associated risks
4.2.3 Evaluating the reliability of a connected object or a mobile application
4.2.4 Using and configuring connected objects and health applications

4.3 Using suitable foundational tools and services and identifying their articulation with other shared records

4.3.1 Knowing the technical doctrine of digital health
4.3.2 Using services associated with the Shared Medical Record (DMP) and My Health Space (MES)
4.3.3 Knowing the various shared records [pharmaceutical record (DP), the Computerized Patient Record (DPI), and the Computerized User Record (DUI), ...]
4.3.4 Knowing and implementing the guidelines for proper use of e-Prescription
4.3.5 Using digital services for coordination and orientation (e-Parcours, Via trajectory)

4.4 Seeking evidence-based health information

4.4.1 Knowing reliable reference sites and recognizing fraudulent ones
4.4.2 Conducting online research (defining the research and the search equation, ...)
4.4.3 Identifying and evaluating the source of information
4.4.4 Identifying disinformation content
4.4.5 Being able to provide users with the right resources (MES)

5. Telehealth

5.1 Mastering regulation and best practices in telehealth

5.1.1 Knowing the legal, regulatory, and conventional framework of telehealth
5.1.2 Knowing the roles and differentiating the responsibilities of different actors in a care process (privacy protection and respect for medical confidentiality, ...)

5.1.3 Being able to differentiate teleconsultation, teleassistance, telesurveillance, teleexpertise, and telecare
5.1.4 Identifying inherent risks in telehealth practice
5.1.5 Identifying ethical and deontological issues

5.2 Practicing telehealth in connection with the care team and in compliance with best practices

5.2.1 Implementing best practices to ensure the quality and safety of care
5.2.2 Personalizing and leading a telehealth medical or care project (design, implementation, evaluation)
5.2.3 Choosing telehealth tools suitable for practice and according to the preferences of patients and users
5.2.4 Informing and supporting the patient and their caregivers effectively
